# Case Report: Adjuvant radiation therapy for cardiac intimal sarcoma with long-term disease control

**DOI:** 10.3389/fonc.2026.1772417

**Published:** 2026-03-05

**Authors:** Ariel Rosa, John G. Roubil, Raj Singh, Navid Fallahi, Andrew Poklepovic, Elisabeth Weiss

**Affiliations:** 1 Department of Radiation Oncology, Massey Comprehensive Cancer Center, Virginia Commonwealth University (VCU) Health System, Richmond, VA, United States; 2 The James Cancer Hospital and Solove Research Institute, The Ohio State University Wexner Medical Center, Department of Radiation Oncology, Columbus, OH, United States; 3 Division of Hematology, Oncology, and Palliative Care, Department of Internal Medicine, Massey Comprehensive Cancer Center, Virginia Commonwealth University (VCU) Health System, Richmond, VA, United States

**Keywords:** adjuvant radiation therapy, cardiac intimal sarcomas, CIS, VMAT, volumetric modulated arc therapy

## Abstract

Cardiac intimal sarcomas (CIS) are rare, aggressive primary cardiac malignancies with limited data guiding adjuvant treatment. We report a case of a 40-year-old woman with bi-atrial CIS who underwent bi-atrial resection and mitral valve replacement, with pathology confirming an MDM2-amplified intimal sarcoma and positive margins (R1). She received four cycles of adjuvant chemotherapy followed by adjuvant radiation therapy (RT) delivered using volumetric modulated arc therapy (VMAT), planned with four-dimensional computed tomography to account for respiratory motion, to a total dose of 60 Gy with a 6-Gy sequential boost. Treatment was well tolerated, with only grade 1–2 acute toxicities that resolved by completion of therapy. At 33 months following completion of RT and 41 months from diagnosis, the patient remains alive and disease-free without evidence of recurrence or significant late toxicity. This case supports the feasibility of incorporating adjuvant RT into a multimodal treatment approach for CIS and suggests that advanced radiation planning techniques may allow safe delivery of curative-intent doses to the heart, although further studies are needed to define its role in this rare malignancy.

## Introduction

Cardiac intimal sarcomas (CIS) are rare, aggressive primary cardiac malignancies arising from the endocardial lining of the heart (1, 2). They represent a distinct subtype of primary cardiac sarcoma and are frequently misdiagnosed as benign cardiac masses, such as myxomas, vegetations, or thrombi, likely contributing to an underestimation of their true incidence (2–4). Because of their rarity, available data are largely derived from case reports and small series.

CIS most commonly affect middle-aged adults and typically involve the left atrium, although right-sided and bi-atrial disease have been reported (5, 6). Clinical presentation is variable, ranging from cardiac symptoms such as dyspnea and palpitations to constitutional symptoms, with some patients remaining asymptomatic until advanced disease (5). Diagnosis is initiated with echocardiography and further refined using advanced imaging modalities, including cardiac magnetic resonance (CMR) imaging and positron emission tomography, which aid in tumor characterization and staging (6–10).

CMR in particular plays a significant role in the evaluation of cardiac masses by providing tissue characterization that improves differentiation of malignant tumors from benign masses and thrombi. Late gadolinium enhancement (LGE) reliably distinguishes tumor from thrombus, as thrombi are typically non-enhancing due to avascularity, whereas malignant tumors demonstrate post-contrast enhancement (11). First-pass perfusion and native T1 mapping further favor malignancy by identifying increased vascularity and elevated T1 values (12, 13). Cine imaging, a type of magnetic resonance imaging (MRI) sequence acquired to capture motion, provides morphologic features suggestive of malignancy, such as infiltrative growth, sessile attachment, polylobate morphology, and pericardial effusion (14, 15). These integrated features achieve high accuracy for predicting malignancy cardiac tumors.

Definitive diagnosis requires histopathologic confirmation (4). CIS are high-grade, pleomorphic sarcomas characterized by immunohistochemical expression of markers such as vimentin, mouse double minute 2 homolog (MDM2), and cyclin-dependent kinase 4 (CDK4), with MDM2 gene amplification on fluorescence *in situ* hybridization serving as a key diagnostic hallmark (6, 16).

Surgical resection remains the cornerstone of treatment, although complete (R0) resection is often challenging due to infiltration of critical cardiac structures (9). Chemotherapy (CHT) and radiation therapy (RT) have been used in select cases, primarily in the adjuvant or unresectable setting, but no standardized treatment paradigm exists (3, 5). Historically, RT has been limited by concerns regarding cardiotoxicity; however, advances in radiation planning and delivery have renewed interest in its role for improving local control in patients with high-risk or incompletely resected disease (3, 5).

## Case presentation

**Figure 1:**
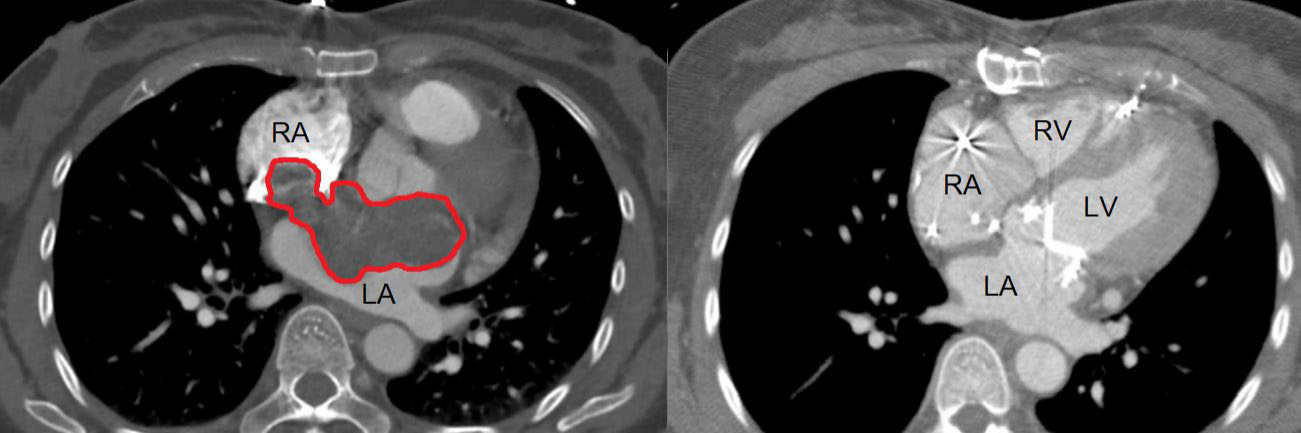
Preoperative (left) and postoperative (right) contrast-enhanced chest CT images on demonstrating a large filling defect (red) corresponding to the tumor mass involving the bilateral atria preoperative imaging, with resolution of the defect following surgical resection on postoperative imaging. LA, left atrium; RA, right atrium; LV, left ventricle; RV, right ventricle.

A 40-year-old woman with no significant past medical history initially presented with 7 months of dyspnea on exertion. Echocardiogram revealed a bi-atrial mass concerning for thrombosis versus neoplasm (e.g., myxoma), an ejection fraction of 55%–60%, moderate mitral regurgitation, moderate to severe mitral stenosis, and mild tricuspid regurgitation. CT of the chest with and without contrast demonstrated cardiac filling defect involving right and left atria and spanning the atrial septum (see **Figure 1**). MRI of the chest without contrast demonstrated a large T2-hyperintense bi-atrial mass, but was otherwise limited by cardiac and respiratory motion and lack of contrast. Patient underwent bi-atrial resection, atrial septal defect repair with a pericardial patch, and mechanical mitral valve replacement. The cardiac mass was removed in a piecemeal fashion, resulting in an R1 resection with positive surgical margins. The procedure was complicated by significant blood loss and third-degree heart block requiring pacing, with the patient ultimately needing a permanent pacemaker. Post-operative echocardiogram revealed an ejection fraction of 55%–60% and no mitral regurgitation or stenosis.

Preliminary surgical pathology was concerning for sarcoma and underwent peer review at a sarcoma reference center. Histopathologic evaluation demonstrated a high-grade pleomorphic spindle cell neoplasm with marked cytologic atypia and mitotic activity. Immunohistochemistry showed patchy positivity for smooth muscle actin and calponin with the absence of epithelial, endothelial, and neural markers, and fluorescence *in situ* hybridization confirmed MDM2 gene amplification, consistent with CIS. The patient was referred for evaluation at a comprehensive cancer center.

Three months following surgical resection, the patient began adjuvant CHT consisting of doxorubicin, ifosfamide, and dexrazoxane, every 3 weeks for four total cycles. Her final course of CHT treatment was complicated by culture-negative septic shock and pancytopenia, from which she made a complete recovery. Otherwise, the patient tolerated CHT well. She was also followed by Signatera tumor-informed ctDNA molecular residual disease (MRD) testing. Immediately prior to initiating adjuvant CHT, her MRD test was positive at a value of 0.19 MTM/mL. Signatera MRD testing cleared following cycle 1 and has remained negative since that time. Three days following her last cycle, post-CHT imaging, including cardiac CT and CT of the abdomen and pelvis, was negative for both local recurrence and metastatic disease. Following systemic therapy, she was referred to radiation oncology.

The patient was simulated in the supine position, head-first, with her arms raised to grasp a wing-board. A contrast-enhanced free breathing 4D-CT scan of the chest was acquired for assessment of respiratory motion management. The presurgical tumor bed (GTV) was contoured, then expanded to an iGTV, which is the gross tumor volume (GTV) expanded to include its internal motion and deformation during the respiratory cycle and other physiologic movements. Contrast was used to better visualize myocardium and cardiac substructures. An additional 5-mm internal target volume (ITV) expansion was used to account for microscopic disease. A 5-mm planning target volume (PTV) expansion was added to account for setup uncertainty. The boost volume (mitral valve, pulmonary valve, and areas of positive margin) was expanded in the same fashion. The iGTV tumor bed volume, ITV, and PTV volumes were 109.19, 206.64, and 322.14 cm^3^, respectively. The boost ITV and PTV were 25.87 and 61.09 cm^3^, respectively.

**Figure 2:**
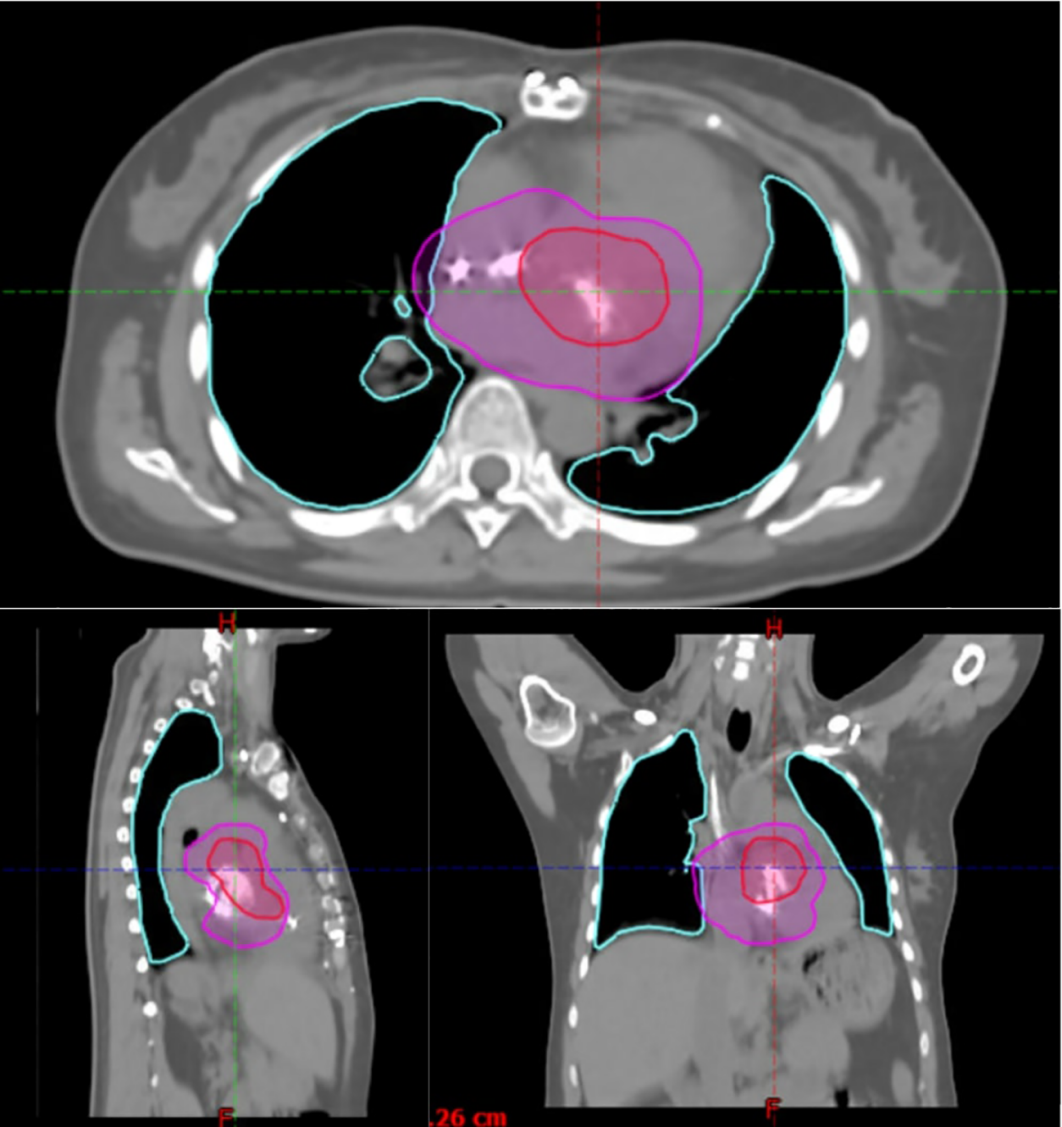
Axial, sagittal, and coronal views of the initial PTV (magenta) and PTV boost volume (red), and bilateral lungs (cyan).

Organs at risk and other structures were contoured on the average CT generated from the 4D CT including heart, lungs, esophagus, and spinal canal as a surrogate for spinal cord, pacemaker, and left anterior descending coronary artery. The surgical bed was treated to 60 Gy in 30 fractions, followed by a sequential 6-Gy (3 fraction) boost to areas concerning for residual disease (see **Figure 2**). Dose was prescribed so that 98% of the PTV and 100% of the ITV received prescription dose. Mean heart dose was 50.95 Gy, dose to 1 cm^3^ of the LAD was 13.5 Gy, mean lung-ITV dose was 13.42 Gy, maximum spinal canal dose was 45.74 Gy, and the pacemaker received a maximum dose of 1.24 Gy.

Treatment was delivered using volumetric modulated arc therapy (VMAT) with 6 MV photons to avoid electron disequilibrium and prevent significant neutron generation in the setting of her pacemaker. *In vivo* dosimetry was used for her first fraction to assess the dose to her pacemaker. Daily pretreatment kV planar imaging and cone-beam CT (CBCT) were used for image guidance. The patient started radiation treatments 1 month following the last cycle of CHT and 6 months following the initial resection.

**Figure 3:**
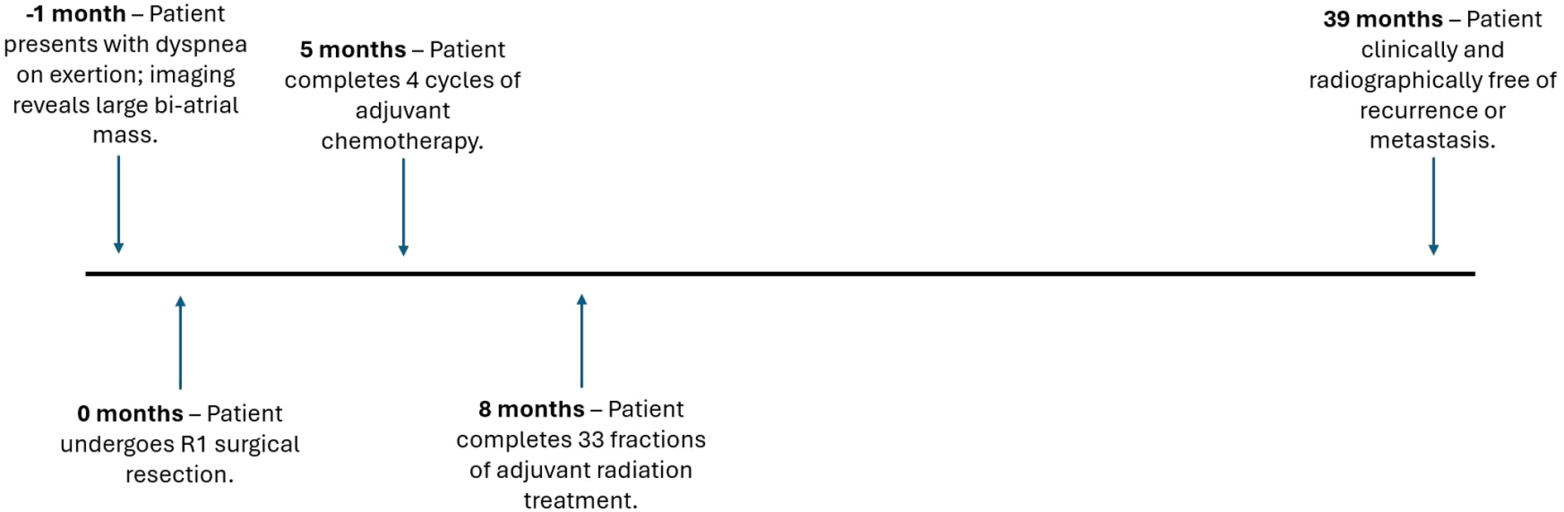
Timeline with significant events relevant to the patient’s case.

During the course of her treatment, she experienced grade 2 (CTCAE V5) dyspepsia during her second week of RT; however, symptoms resolved with the use of an over-the-counter proton pump inhibitor. At week 3, she was noted to have grade 1 esophagitis and grade 1 dysphagia, both of which resolved after the end of RT. She had a post-treatment cardiac CT and CT abdomen and pelvis 2 weeks after completing RT, and both were negative for progression. Follow-up imaging with cardiac CT and CT abdomen and pelvis was completed every 3 months and continues to be negative 33 months post completion of RT. Cardiac MRI at 6 and 18 months post-RT was also negative for recurrence or new structural or functional cardiac changes, and she continues to be followed with annual cardiac MRIs. Transthoracic echocardiogram at 28 months post-treatment showed an EF of 40%–45% (compared to 55%–60% pretreatment), physiologic mitral regurgitation, severe aortic and tricuspid regurgitation, and no coronary artery calcifications. The patient is closely followed by cardiology and is seen every 6 months. At 33 months after completing treatment, she remains disease-free with no clinically significant adverse toxicity attributable to RT. **Figure 3** is a timeline of important events pertaining to this patient’s case.

## Discussion

The frequent misdiagnosis of CIS underscores the importance of advanced imaging, particularly CMR. CMR improves diagnostic accuracy over echocardiography and CT by integrating morphologic and functional evaluation and has been shown to reliably distinguish malignant tumors from benign masses (15). In addition to guiding diagnosis and surgical planning, CMR is valuable for post-treatment surveillance, as in this case.

In this case, adjuvant RT was pursued to improve local control in the setting of non-metastatic disease and microscopically positive surgical margins. Although CIS are relatively chemosensitive and the patient received anthracycline-based CHT, recurrence has been reported even in the setting of adjuvant CHT, and local and distant failure remains a dominant pattern, particularly when complete resection is not achievable (17, 18). RT was therefore incorporated as part of a multimodal, curative-intent approach to address residual microscopic disease. Evidence supporting adjuvant RT in this setting remains limited, which was the primary motivation for presenting this case.

High-dose RT to the heart carries potential risks of long-term cardiotoxicity, including cardiomyopathy, coronary artery disease, valvular dysfunction, and conduction abnormalities. However, modern techniques such as VMAT and 4D CT-based planning allow improved target conformity and sparing of critical cardiac structures. In this case, careful planning and motion management enabled safe delivery of curative-intent doses with acceptable exposure to the heart and other organs at risk, and extended follow-up has demonstrated preserved cardiac function without clinically significant late toxicity.

In a review of CIS cases in the literature, a total of 17 additional CIS cases involving RT were reported beyond our own case (see **Table 1**). Patients had a mean age of 54 years (range: 35–70). The cohort was predominantly male (10/17, 58%), with most tumors located in the left atrium (10/17, 58%). Surgical resection was performed in most patients (15/17, 88%). Among the two patients who did not undergo resection, one received RT following non-response to CHT, while the other was treated with RT alone. Among those who underwent surgery, resections were reported as either R1 (microscopically positive margins), R2 (macroscopically positive margins), or without further margin specification. No case explicitly documented complete resection with negative margins (R0).

**Table 1:** Review of cases that incorporated radiotherapy in the management of CIS.

Case report/series	Patient	Surgery	Chemotherapy	Radiotherapy	Follow-up/Outcome
Diamond et al. (19)	51F with LA mass	R1 resection	None	Adjuvant proton therapy, 66 Gy in 37 Fx	Not reported
Aviel et al. (20)	35M with RV, main PA, and right PA mass	None (patient unresectable)	Adjuvant	HDR brachytherapy (trans-femoral access), 20 Gy in 1 Fx	Symptomatic and radiographic improvement at 10 months; no RT toxicity; developed widespread metastasis; expired 38 months after initial diagnosis
Alam et al. (21)	55F with LA mass	R1 resection	Palliative, for metastasis following RT	Palliative CyberKnife, details not reported	Primary site recurrence + oligometastatic disease at 20 months; expired 3 years after diagnosis
Holzhauser et al. (22)	70F with LA mass	R2 resection	Palliative, for metastasis following RT	Adjuvant RT to PA stem, details not reported	Discovered to have widespread metastasis at 6 weeks post-resection; expired (interval not reported)
Lloyd et al. (23)	57M with LA mass	R1 resection	None	Adjuvant RT to tumor bed; details not reported	Developed probable metastatic abdominal disease; expired 14 months after initial diagnosis
Pomp et al. (24)	58M with LA mass	R1 resection	None	Adjuvant MRI-guided stereotactic adaptive RT, 60 Gy in 12 Fx	Reported to have stable disease on CT imaging 6 months post-RT
Al-Hajri et al. (25)	59M with RVOT mass	R1 resection	None	Adjuvant IMRT to post-op bed, 60 Gy in 30 Fx	Tolerated RT well; no evidence of recurrence or metastasis on CT imaging 3 months following treatment
Shah et al. (26)	65M with recurrent PA/RVOT mass following R2 resection	R2 resection (for primary)	Adjuvant	Salvage IMRT to tumor, 64 Gy in 32 Fx	Symptomatic and radiographic improvement on 5-month post-RT CT scan; long-term outcome not reported
Al Umairi et al. (27)	55M with RVOT and main pulmonary trunk mass	Resection; type not specified	Adjuvant	Adjuvant VMAT; further details not reported	Follow-up CT imaging at 6 months post-RT without evidence of recurrence or metastasis
Romanowska et al. (28)	47M with RV mass	R1 resection	None	Adjuvant IMRT with SIB, 66 Gy in 33 Fx	RT well-tolerated; disease-free 32 months post-RT
	57M with LA mass	R2 resection (following local recurrence after R1 resection)	Adjuvant	Adjuvant IMRT with SIB, 66 Gy in 33 Fx	RT well-tolerated; complete remission of LA mass, then developed retroperitoneal metastasis 1 year following RT s/p resection and CHT; disease control 4 years post-RT
Spartalis et al. (29)	41F with LA mass	R2 resection	None	Adjuvant RT, details not reported	No signs of recurrence on echocardiogram 9 months post-RT
Chiarelli et al. (30)	50M with LA mass	R1 resection	Adjuvant	Adjuvant RT, 50 Gy + 10 Gy boost in 30 Fx to mediastinum	No progression of primary; develop small bowel metastasis 18 months post-RT s/p resection, CHT, and RT; no evidence of disease at last follow-up (interval not reported)
Moeri-Schimmel et al. (31)	58F with LA mass	R2 resection	Palliative, for metastasis following RT	Adjuvant VMAT, 59.4 Gy in 33 Fx	PET/CT 3 months post-RT showed multiple distant metastases; expired 13 months following initial diagnosis
Supel et al. (32)	36F with pulmonary trunk and right PA mass	R2 resection	Adjuvant	Adjuvant RT, details not reported	Not reported
Awoyemi et al. (33)	68F with RV mass	None (patient unresectable)	None	Palliative RT, 30 Gy total dose, further details not reported	Disease progression with worsening symptoms; patient sought hospice care; further details not reported
Ballout et al. (34)	35M with LA mass with brain metastasis	Resection; type not specified	Adjuvant	Adjuvant RT, details not reported	Not reported
Our case	40F with bi-atrial mass	R1 resection	Adjuvant	Adjuvant VMAT, 60 Gy (in 30 Fx) to presurgical tumor volume with 6 Gy (in 3 Fx) sequential boost to high-risk areas	No clinical or radiographic evidence of disease 28 months post-RT; no clinically significant adverse toxicity from RT

CHT, chemotherapy, CT, computed tomography, Fx, fraction, Gy, gray, HDR, high dose rate, IMRT, intensity-modulated radiation therapy, LA, left atrium, MRI, magnetic resonance imaging, PA, pulmonary artery, RA, right atrium, R1, resection with microscopic positive margins, R2, resection with macroscopic positive margins, RT, radiation therapy, RV, right ventricle, RVOT, right ventricular outflow tract, SIB, simultaneous integrated boost, VMAT, volumetric modulated arc therapy.

CHT was employed in 10 of the 17 cases (58%); 7 received adjuvant CHT, while 3 were treated in the palliative setting for metastatic disease following adjuvant RT. The remaining 7 patients did not receive CHT. RT dosing varied across cases, ranging from a single 20-Gy fraction to 66 Gy delivered in 37 fractions. Most reported doses were 60–66 Gy delivered over 30–33 fractions. RT modalities utilized were primarily VMAT or intensity-modulated radiation therapy (IMRT) in cases where the technique was specified. Other select cases implemented proton therapy (*n* = 1), endovascular high-dose rate (HDR) brachytherapy (*n* = 1), MRI-guided stereotactic adaptive RT (*n* = 1), and stereotactic body RT (SBRT) via CyberKnife (*n* = 1). Several reports did not provide RT details.

Patient outcomes were heterogeneous, though a trend toward poor prognosis was noted. Multiple cases described disease progression and patient death within months to a few years following diagnosis. Of the 17 patients reviewed, 5 (29%) had expired due to disease progression at the time of reporting, underscoring the aggressive clinical course observed in a subset of cases. In contrast, among the reported outcomes, local control was achieved in only 6 cases (35%), as evidenced by descriptions such as stable disease, tumor regression, or absence of recurrence on follow-up imaging. The patients who achieved local control or were disease-free at follow-up generally had an R1 resection followed by adjuvant high-dose RT. Five out of the six (83%) cases achieving local control specifically mention RT doses in the 60–66 Gy range similar to our own case, often with modern conformal techniques, consistent with curative intent dosing. The other case did not disclose RT dosing details.

Among the reported cases, 6 (35%) demonstrated evidence of metastatic disease following RT. In patients where metastases occurred, reported sites of spread were primarily bone (e.g., femur and humerus) or abdomen (e.g., small bowel and retroperitoneal space). RT was generally well tolerated, with no reported toxicity attributable to radiation despite often high doses to large volumes of the heart such as in our case.

Interpretation of these findings is limited by significant heterogeneity and incomplete reporting across the case reports. Many publications lacked comprehensive details regarding treatment modalities, such as the specific radiation techniques, treatment volumes, and timing of RT in relation to surgery. Follow-up intervals and clinical outcomes were also inconsistently reported, making it difficult to assess disease progression or treatment durability across cases. Additionally, data on radiation-associated toxicity were largely absent, limiting the ability to evaluate treatment tolerability. These limitations highlight the need for more standardized and detailed documentation in future reports to better inform the role of RT in managing CIS.

## Conclusion

Overall, RT may offer symptomatic benefit and contribute to local control in select patients, particularly in the setting of non-metastatic, margin-positive disease where the risk of recurrence remains high despite CHT. Its role in improving long-term survival remains unclear due to the paucity of data. Further investigation is warranted to better define the role of RT in the management of CIS, including optimal radiation modalities, dosing strategies, target volumes, strategies to mitigate potential cardiotoxicity, and long-term clinical outcomes. Surgical resection remains the standard of care for these patients; however, multidisciplinary decision-making remains essential in tailoring treatment strategies for this rare malignancy. Modern radiation techniques allow the safe delivery of curative-intent therapy with acceptable cardiac risk. In this multidisciplinary context, advanced cardiac imaging, particularly CMR, plays a crucial role in improving diagnostic accuracy, informing treatment planning, and enabling longitudinal surveillance. Tumor-informed ctDNA is an emerging technology that can track patient outcomes and may help inform treatment decisions in the future.

## Patient perspective

The patient expressed satisfaction with her treatment course and outcome. She was appreciative of the multidisciplinary care she received and reported a positive overall experience. The patient provided informed consent for publication of this case report and expressed gratitude for her care and recovery.

## Data Availability

The original contributions presented in the study are included in the article/supplementary material. Further inquiries can be directed to the corresponding author.
